# Simulation-Based Training for Emergency Department Flow Management: A Dual-Site Experience With GridlockED in France

**DOI:** 10.7759/cureus.105250

**Published:** 2026-03-15

**Authors:** Edouard Lansiaux, Evelyne Guerif Dubreucq, Teresa M Chan

**Affiliations:** 1 Emergency Medicine, Lille University Hospital, Lille, FRA; 2 Emergency Medicine, Hôpital Tenon, Assistance Publique-Hôpitaux de Paris, Paris, FRA; 3 Emergency Medicine, Toronto Metropolitan University, William Osler Health Systems, Brampton, CAN

**Keywords:** crisis leadership, emergency department, flow management, gridlocked, simulation, team communication

## Abstract

Background

Emergency department overcrowding and flow management remain critical challenges worldwide, demanding not only clinical expertise but also robust non-technical skills such as teamwork, communication, and systems thinking.

Material and methods

This program evaluation describes two workshops conducted during major French medical conferences in 2025, using the GridlockED tabletop simulation game as an interactive educational tool for healthcare professionals. A total of 46 participants with diverse professional backgrounds and experience levels completed post-session surveys assessing satisfaction, perceived realism, language accessibility, and self-reported learning gains across five competencies related to emergency department flow management.

Results

Results indicated high overall satisfaction and meaningful perceived learning, particularly in identifying operational barriers, though team communication emerged as the lowest-rated skill. The English-language format represented a moderate barrier for French-speaking participants, highlighting the need for linguistic adaptation.

Conclusions

These findings support the potential of tabletop simulation games as accessible, scalable training tools for enhancing emergency department flow management competencies in non-English-speaking healthcare contexts.

## Introduction

Emergency departments (EDs) worldwide face persistent challenges of overcrowding, delayed patient throughput, and constrained resources. These factors contribute to increased waiting times, reduced quality of care, and heightened stress among healthcare teams. Effective flow management - coordinating patient movement from arrival to discharge while optimizing available resources - is therefore a cornerstone of modern ED practice. Beyond clinical expertise, it demands strong leadership, situational awareness, efficient communication, and the ability to prioritize under pressure.

Traditional training methods for flow management often rely on observational learning or retrospective case reviews, which may limit active skill development. Simulation-based training offers a dynamic alternative, creating an immersive environment where participants can make decisions, experience consequences, and reflect on their performance in real time [1]. While high-fidelity manikin-based simulations are widely used for acute clinical scenarios, tabletop simulation games provide a complementary, cost-effective, and logistically accessible format to train complex non-technical skills such as teamwork, coordination, and systems thinking [2].

GridlockED is one such tabletop simulation tool [3]. It challenges participants to manage a busy ED using a realistic game board, patient cases, and time-based constraints, replicating the pressures and unpredictability of real-world emergency care. The game promotes learning through interactive problem-solving, requiring participants to adapt to evolving situations, allocate limited resources, and maintain patient safety. Previous studies have demonstrated that GridlockED is both enjoyable and educational for medical trainees, with participants reporting meaningful learning about health systems, communication, and multipatient environment management [4,5]. Observational studies have further identified teachable moments embedded within gameplay, including resource prioritization, human resource management, and patient flow optimization [6]. These documented teachable moments directly informed the selection of the five competency domains targeted in our workshop design.

Although GridlockED has been validated as an educational tool in North American training programs, no prior study has evaluated its use in a French-language, non-North American healthcare context. This represents a meaningful gap: cultural, linguistic, and organizational differences between healthcare systems may influence how simulation tools are perceived, experienced, and integrated into professional development. To our knowledge, this is the first evaluation of GridlockED conducted simultaneously across two distinct French conference settings, with a sample predominantly composed of nursing professionals - a population underrepresented in the existing GridlockED literature.

In 2025, we conducted two GridlockED workshops in France: one during the French Society of Emergency Medicine Congress (Congrès de la Société Française de Médecine d'Urgence) and another during the French Society of Simulation Congress (Congrès de la Société Française de Simulation). Each event gathered healthcare professionals with varying levels of experience, providing an opportunity to evaluate the game's educational impact across diverse audiences.

This program evaluation pursued three distinct objectives: 1) To measure participant satisfaction with the GridlockED tabletop simulation game across both workshop settings; 2) To quantify self-reported learning gains across five pre-defined ED flow management competencies: understanding ED function, facilitation tools, barrier identification, team communication, and task allocation; 3) To identify qualitative barriers and facilitators to the use of GridlockED in the French-language healthcare context.

## Materials and methods

We conducted a program evaluation of two workshops held at French-language medical conferences between June 4 and June 12, 2025. Session 1 took place at the French Society of Emergency Medicine Congress (SFMU); session 2 was held at the French Society of Simulation Congress (SoFraSims/SIMCUP). We adopted a logic model program evaluation framework [7], operationalized as follows: inputs (facilitator expertise, game materials, participant diversity), activities (gameplay, facilitated debrief), outputs (survey responses), and short-term outcomes (perceived learning gains across five competencies).

Workshop description

Each workshop began with a 15-minute structured introduction covering ED flow principles and GridlockED game mechanics. Participants were divided into small interprofessional teams of three to five players per table, each managing a simulated ED. Total session duration was approximately 90 minutes, including gameplay and debrief. The version of GridlockED used was the standard commercial edition published by Tsoy et al. [3].

Facilitation was led by the first author (E.L.), an emergency physician with prior experience facilitating simulation-based education. Facilitators ensured rule compliance, guided decision-making, and supported participant engagement throughout gameplay. A standardized debrief structure was employed following gameplay, guided by the principle of reflective debriefing as formative assessment [8]. The game simulated realistic ED constraints: limited beds, resource allocation dilemmas, and unexpected patient influxes.

The workshop was designed to develop five competencies relevant to effective ED flow management, each of which has been documented as a teachable moment in prior GridlockED research [6]: 1) understanding ED function; 2) identification of facilitation tools for flow management; 3) identification of barriers to flow management; 4) team communication; 5) task allocation.

Survey tool

A four-point Likert scale (1 = strongly agree, 4 = disagree) was used, alongside open-ended feedback prompts. The questionnaire was offered to participants via an anonymizing survey platform (Wooclap, Brussels, Belgium). A copy of the questionnaire is provided in Appendix 1 (Table 4). To structure this evaluation, we followed the Standards for QUality Improvement Reporting Excellence in Education (SQUIRE-EDU) reporting framework and its associated checklist.

The questionnaire was adapted from the previously published GridlockED Game Play Survey developed by Tsoy et al. [3]. It was not independently re-validated for the French context; as such, no Cronbach's alpha coefficient can be reported for the current adaptation. This constitutes a methodological limitation acknowledged in the Limitations section below.

Research ethics

According to French law (Loi Jardé), this type of program evaluation study does not require authorization from an Institutional Review Board or ethics committee. Participant responses were collected anonymously.

Data collection

After gameplay, participants completed surveys rating their satisfaction, perceived realism, language barriers, and learning gains across the five competencies: understanding ED function, facilitation tools, barrier identification, team communication, and task allocation.

Data analysis

Participant responses were collected using a Likert scale, with each item rated from 1 (most favorable) to 4 (least favorable). For each workshop session and each item, the mean score was calculated by averaging the numerical ratings across all participants. This approach allowed the quantification of overall trends in satisfaction and perceived learning, providing a continuous measure of central tendency for each evaluated aspect of the game.

To facilitate interpretation and comparison across items, the mean scores were converted into percentages, representing their equivalence on a 0-100% scale. In this conversion, lower mean scores (closer to 1) correspond to higher percentages, indicating stronger agreement or better perceived performance, whereas higher mean scores (closer to 4) correspond to lower percentages, reflecting less favorable responses.

The data were summarized for each session separately, as well as globally across sessions, to capture both session-specific variations and overall trends. Between-group comparisons were performed using the Mann-Whitney U test for ordinal Likert-scale data and Fisher's exact test for categorical variables, given the small sample sizes and non-normal distributions. A p-value of <0.05 was considered statistically significant. The combination of mean scores and percentage equivalence provides a clear, interpretable representation of participant responses while maintaining fidelity to the original Likert-scale ratings.

## Results

Across both workshops, a total of 46 participants completed the post-session survey. Workshop 1, held during the French Society of Emergency Medicine Congress, included 18 participants whose professional experience in healthcare ranged from less than one year to two decades, with a mean of 9 years. Workshop 2, conducted during the French Society of Simulation Congress, involved 28 participants, whose experience ranged from complete novices (0 years) to 56 years in practice, with a mean of 12.6 years.

As shown in Table 1, nurses represented the majority of participants across both workshops (83.3% in workshop 1; 85.7% in workshop 2), with no significant difference in professional distribution between sessions (Fisher's exact test, p=0.312). Years of professional practice are summarized in Table 2; no significant between-group difference was observed (Mann-Whitney U=236.5, p=0.847).

**Table 1:** Professional distribution of participants across workshops Percentages indicate the proportion within each workshop. The p-value of 0.312 (Fisher's Exact Test) indicates no significant difference in the professional makeup of the two groups.

Profession	Workshop 1 (n=18)	Workshop 2 (n=28)	Global (N=46)
Nurse	15 (83.3%)	24 (85.7%)	39 (84.8%)
Nursing assistant	0 (0%)	2 (7.1%)	2 (4.3%)
Senior doctor	0 (0%)	1 (3.6%)	1 (2.2%)
Medical student	0 (0%)	1 (3.6%)	1 (2.2%)
Health executive	1 (5.6%)	0 (0%)	1 (2.2%)

**Table 2:** Years of professional practice Between-group comparison by Mann-Whitney U test (U=236.5, p=0.847).

Group	Min	Max	Median	Mean	± SD	p-value
Workshop 1	3	20	8	9.00	6.07	
Workshop 2	0	56	7	12.60	13.79	
Global	0	56	7.5	11.20	11.35	0.847

Overall participant satisfaction with the GridlockED workshops was high, with a global mean score of 1.29 ± 0.59 on the 1-4 Likert scale, where 1 signifies strong agreement or satisfaction. Appreciation was significantly greater among attendees at the French Society of Simulation Congress (workshop 2), who reported a mean satisfaction score of 1.00 ± 0.00. In comparison, participants at the French Society of Emergency Medicine Congress (workshop 1) reported a mean of 1.57 ± 0.73 (Mann-Whitney U=148.0, p=0.003).

The English-language format of the game was identified as a moderate barrier. The overall cohort rated language transferability at 2.00 ± 1.07. The difference between workshop 1 (2.14 ± 1.12) and workshop 2 (1.86 ± 0.99) was not statistically significant (U=221.0, p=0.536).

Perceptions of the game's realism were moderate overall (1.64 ± 0.89). Workshop 2 participants rated the experience significantly more favorably (1.14 ± 0.35) than their workshop 1 counterparts (2.14 ± 0.99; U=112.5, p<0.001). Complete survey response data are presented in Table 3.

**Table 3:** Survey responses by workshop session with between-group comparisons Likert scale: 1 = strongly agree (most favorable) to 4 = disagree (least favorable). Between-group comparisons were performed using the Mann-Whitney U test. * indicates p<0.05.

Item	W1 mean	W1 SD	W2 mean	W2 SD	Global mean	U statistic	p-value
Game satisfaction	1.57	0.73	1.00	0.00	1.29	148.0	0.003*
Language transferability	2.14	1.12	1.86	0.99	2.00	221.0	0.536
Game realism	2.14	0.99	1.14	0.35	1.64	112.5	<0.001*
Emergency working	2.13	1.17	1.88	1.27	2.00	218.0	0.491
Helping factors identification	2.25	0.97	1.13	0.33	1.69	108.0	<0.001*
Hindering factors identification	1.67	0.75	1.00	0.00	1.33	168.0	0.008*
Team communication	2.33	1.24	2.00	1.41	2.17	220.5	0.527
Task distribution	2.00	1.00	1.25	0.66	1.63	171.5	0.024*

As shown in Table 3, game satisfaction (1.29 ± 0.59) and hindering factors identification (1.33 ± 0.62) received the most favorable global ratings, with mean scores closest to 1. Helping factors identification (1.69 ± 0.92) and game realism (1.64 ± 0.89) showed more variation between sessions, both reaching statistical significance (p<0.001). Language transferability (2.00 ± 1.07), emergency working (2.00 ± 1.22), team communication (2.17 ± 1.34), and task distribution (1.63 ± 0.93) received intermediate scores, clustering around 2 on the Likert scale. The global means reflect moderate to high overall satisfaction, with most criteria falling between 1.3 and 2.2 on the 1-4 scale.

## Discussion

The GridlockED workshops demonstrated meaningful educational benefits, particularly in enhancing systemic awareness and process optimization skills in emergency department contexts. Participants reported that the game effectively helped them identify operational barriers, with barrier recognition consistently highlighted as a key learning outcome. The second session showed greater gains in using facilitation tools for patient flow and task allocation. While participants experienced moderate improvements in understanding ED functioning, gains in team communication were more limited, indicating a need for complementary strategies to strengthen interprofessional collaboration.

Between-session differences in satisfaction and realism ratings are grounded in our data (Tables 2 and 3) and warrant careful interpretation. Workshop 2 participants, attending a simulation-focused congress, were likely more familiar with simulation-based pedagogy, which may have facilitated engagement and immersion. The broad distribution in professional experience across both sessions - blending highly seasoned practitioners with relative newcomers - further contributed to heterogeneous learning dynamics and may have influenced the nature of the feedback received. We interpret these differences as reflecting genuine variations in audience composition rather than shortcomings in the game itself.

We additionally hypothesize that contextual factors beyond audience experience may have shaped engagement: familiarity with local ED terminology, the facilitation style of the session leader, and participants' prior exposure to tabletop formats could each play a role. Future studies should systematically examine these moderating variables to better understand what drives session-level variability.

These findings are consistent with those reported by Chan et al. [4], who conducted GridlockED workshops across Canadian training programs and found that 72 participants rated the game highly for both enjoyment and educational value (mean ratings of 6.53 and 6.17 out of 7, respectively). In their study, participants similarly identified key learning points related to prioritization, resource utilization, and multipatient management. Notably, Chan et al. highlighted task switching as a core competency developed through GridlockED gameplay, a finding that resonates with our observation that task distribution was among the more favorably rated competencies, particularly in workshop 2. The GAME study by Chan et al. [6] further demonstrated through direct observation that experienced practitioners and trainees could identify specific teachable moments within GridlockED, including resource management, patient flow optimization, and human resource deployment, though deviations from real-world ED processes were also noted, underscoring the importance of structured facilitation and debriefing.

Our findings also align with broader evidence on the educational potential of serious games in emergency medicine. Chon et al. [9] evaluated EMERGE, a virtual emergency department serious game, among 140 medical students in Germany and demonstrated significant gains in both declarative and procedural knowledge (p<0.001), alongside high student satisfaction. Their observation that students in lower semesters showed greater knowledge gains and more positive impressions parallels our finding that participant engagement and perceived realism varied with group composition and experience level. Similarly, Abensur Vuillaume et al. [10] described the development of an educational escape game for emergency medicine in France, emphasizing the value of gamified approaches for teaching teamwork and diagnostic reasoning in multidisciplinary settings. Their study reinforced the importance of structured debriefing and facilitator involvement - elements also central to our GridlockED workshops.

The consistent finding that team communication was the lowest-rated skill underscores a limitation that is not unique to GridlockED but common in many simulation formats when communication is not explicitly targeted. A systematic review by Blackmore et al. [11] on simulation-based education for communication skills found that while simulation-based training can improve team communication, the gains are often short-lived and require sustained, repeated training sessions to be maintained. Furthermore, the review noted that in situ simulation approaches may produce more durable improvements compared to off-site interventions. Future iterations of our GridlockED workshops should integrate structured communication strategies, such as SBAR (Situation, Background, Assessment, Recommendation) or closed-loop communication, into gameplay and debriefing [12,13]. The TeamSTEPPS framework, developed by the Agency for Healthcare Research and Quality, provides an evidence-based set of teamwork tools that could be readily incorporated into the GridlockED format to enhance interprofessional communication outcomes [14].

Regarding realism, the observed disparity between workshop 1 and workshop 2 can be interpreted in light of both physical and psychological fidelity. Rudolph et al. [8] and Kolbe et al. [15] have highlighted that perceived realism in simulation is influenced not only by physical fidelity but also by psychological fidelity - the degree to which the simulation engages participants in authentic cognitive and emotional processes. The higher realism scores in workshop 2 are consistent with this framework: participants with prior simulation experience are more likely to engage psychologically with the game's constraints. We speculate that tailoring scenarios to the local ED environment and incorporating familiar terminology could further enhance immersion, though this requires prospective evaluation.

Language barriers emerged as a non-negligible limitation, with the overall cohort rating this item at 2.00 on the four-point scale. This finding is consistent with the broader literature on cross-cultural adaptation of educational tools in healthcare. Skjerve et al. [16] demonstrated that language barriers in simulation exercises can be addressed through iterative adaptation and bilingual resource development, resulting in enhanced learning outcomes for non-native speakers. Translation of materials, or provision of bilingual resources, could significantly improve accessibility and learning outcomes. Given the strong engagement despite this barrier, it is reasonable to expect even higher satisfaction and learning gains once the game is fully adapted to the French context. Charnetski et al. [17] conducted a scoping review on cultural considerations in simulation curriculum adaptation, emphasizing the importance of contextual and linguistic adaptation for successful international implementation of simulation tools.

Participants' enthusiasm for GridlockED highlights its potential for wider adoption. Its scalability and low-resource design make it suitable for repeated training, multidisciplinary exercises, and inter-hospital competitions. These competitions could allow multiple ED teams to engage in the same scenarios, compare strategies, and share best practices, fostering cross-institutional learning, innovation, and collaboration. Structured debriefs would enable benchmarking, identification of transferable practices, workflow standardization, and stronger regional emergency care networks. The tabletop format offers distinct advantages over high-fidelity simulation in terms of cost, portability, and scalability, as previously noted in the disaster preparedness literature, where tabletop exercises serve a complementary role to full-scale simulations [7].

Limitations

This study has several limitations. First, the sample size was relatively small (N=46), limiting the generalizability of findings. Second, the use of self-reported measures introduces the possibility of social desirability bias. Third, the absence of a control group and pre-post assessment design precludes causal inferences about learning outcomes. Fourth, the survey instrument was adapted from the original GridlockED Game Play Survey [3] and was not independently re-validated for the French-language context; no Cronbach's alpha or other measure of internal consistency can therefore be reported for this adaptation. Fifth, facilitator standardization was not formally operationalized beyond the experience of the lead facilitator, which may limit reproducibility. Finally, the predominantly nursing composition of our sample may not reflect the full interprofessional diversity of ED teams. Future studies should include larger, more diverse samples, employ validated and locally adapted assessment instruments, incorporate facilitator training protocols, and include longitudinal follow-up to evaluate skill retention and transfer to clinical practice.

## Conclusions

GridlockED demonstrated perceived educational value as a learning platform for enhancing ED flow management skills among French-language healthcare professionals with diverse backgrounds, with participants reporting high satisfaction and meaningful self-reported learning gains, particularly in identifying operational barriers to patient flow. These findings should be interpreted as evidence of perceived educational benefit rather than demonstrated learning effectiveness, given the absence of a control group and validated pre-post measures. Language barriers and communication skill gaps remain areas for targeted improvement, though these limitations are surmountable through contextual adaptation, translation, and structured communication training. Future research should focus on longitudinal evaluation of skill retention, the development of a validated French-adapted survey instrument, and the comparative effectiveness of tabletop simulation versus other training modalities in this domain.

## Appendices

**Table 4:** Original French questionnaire and corresponding English translation used for program evaluation. The questionnaire was adapted from the GridlockED Game Play Survey [3]. Scale Legend: Items were rated on a 4-point Likert scale: 1 = Tout à fait d’accord (Strongly agree); 2 = D’accord (Agree); 3 = Pas d’accord (Disagree); 4 = Pas du tout d’accord (Strongly disagree).

French (Original)	English (Translation)
Information démographique	Demographic Information
Depuis combien d’années exercez-vous votre activité professionnelle ?	For how many years have you been practicing your professional activity?
Quelle est votre profession ?	What is your profession?
Évaluation de l'expérience	Experience Evaluation
Avez-vous aimé jouer au Serious Game ?	Did you enjoy playing the Serious Game?
Est-ce que l’anglais était un frein pour vous ?	Was the English language a barrier for you?
Est-ce que le plateau et les éléments correspondaient à votre lieu de travail ?	Did the board and elements correspond to your workplace?
Objectifs d'apprentissage	Learning Objectives
GridlockED vous a appris le fonctionnement d’un service d’urgences.	GridlockED taught you how an emergency department functions.
GridlockED vous a appris les outils facilitateurs à la bonne gestion du flux.	GridlockED taught you the tools that facilitate good flow management.
GridlockED vous a appris les freins à la bonne gestion du flux.	GridlockED taught you the barriers to good flow management.
GridlockED vous a appris à mieux communiquer en équipe.	GridlockED taught you how to better communicate as a team.
GridlockED vous a appris à mieux répartir les tâches.	GridlockED taught you how to better distribute tasks.
Questions ouvertes	Open-Ended Questions
De manière générale, GridlockED vous a apporté des connaissances sur quel aspect en particulier ?	In general, on which aspect in particular did GridlockED provide you with knowledge?
Citez 3 choses que vous avez appris grâce au Serious Game.	List 3 things you learned thanks to the Serious Game.
